# Industry 4.0 Quantum Strategic Organizational Design Configurations. The Case of 3 Qubits: Two Report to One

**DOI:** 10.3390/e23040426

**Published:** 2021-04-03

**Authors:** Javier Villalba-Diez, Juan Carlos Losada, Rosa María Benito, Daniel Schmidt

**Affiliations:** 1 Fakultät Management und Vertrieb, Campus Schwäbisch Hall, Hochschule Heilbronn, 74523 Schwäbisch Hall, Germany; 2 Complex Systems Group, Universidad Politécnica de Madrid, Av. Puerta de Hierro 2, 28040 Madrid, Spain; juancarlos.losada@upm.es (J.C.L.); rosamaria.benito@upm.es (R.M.B.); 3 Department of Business Intelligence, Escuela Técnica Superior de Ingenieros Industriales, Universidad Politécnica de Madrid, 28006 Madrid, Spain; Daniel.Schmidt@saueressig.de; 4 Matthews International GmbH, Gutenbergstraße 1-3, 48691 Vreden, Germany

**Keywords:** quantum strategic organizational design, Industry 4.0, quantum circuits

## Abstract

The goal of this work is to explore how the relationship between two subordinates reporting to a leader influences the alignment of the latter with the company’s strategic objectives in an Industry 4.0 environment. We do this through the implementation of quantum circuits that represent decision networks. In fact, through the quantum simulation of strategic organizational design configurations (QSOD) through five hundred quantum circuit simulations. We conclude that the alignment probability of the leader is never higher than the average alignment value of his subordinates, i.e., the leader never has a better alignment than his subordinates. In other words, the leader cannot present asymptotic stability better than that of his subordinates. The most relevant conclusion of this work is the clear recommendation to the leaders of Industry 4.0 not to add hierarchical levels to their organization if they have not achieved high levels of stability in the lower levels.

## 1. Introduction

Strategic planning in an organization, according to Grant [1], involves the beginning of the strategic process: “A dialogue through which knowledge is shared and consensus and commitment toward action and results are achieved.” This dialogue, previously described as Nemawashi [2] or “catch-ball” [3] by scholars, provides a balance of forces, sometimes delicate, between the interests of the different organizational agents [4]. Operating under a strategic organizational design paradigm [5,6], the interplay of these interdependent organizational elements forms complex hierarchical networks [7] and supports decision making in order to achieve, ideally, a coordination of efforts in pursuit of the organization’s strategic objectives called organizational alignment. Such alignment efforts can occur in different organizational environments, although in this paper the authors focus on complex networked cyber-physical systems in an Industry 4.0 context.

The term Industry 4.0 has gained a lot of traction since it was first publicized [8], stating the need for a paradigm shift towards a less centrally controlled manufacturing structure. It is seen as the fourth industrial revolution with the first three being mechanization through steam power, mass production through electrically operated engineering and finally the digital revolution through the integration of electronics and IT. Industry 4.0 ought to enable a bigger autonomy of the production, as the technology gets more interconnected and machines are able to exert influence on each other creating a cyber-physical system. The term cyber-physical system in the context of Industry 4.0 refers to the tight conjoining of and coordination between computational and physical resources. The impact on the development of such systems is a new paradigm of technical systems based on collaborative embedded software systems [9].

Approaches to qualitatively model organizational alignment have been proposed by several scholars [10,11,12,13,14,15,16]. Less common are approaches that allow quantifying the organizational alignment [2], where the alignment status of each node is known at each discrete time interval. The NEMAWASHI approach, based on genetic algorithms, is, however, computationally very expensive and therefore difficult to implement in practice. While the computation of the alignment state of the entire network is theoretically possible with this method, in practice it is a challenge that leads to an exponential increase in computational time with increasing network size. For this reason, there is an urgent need to provide organizational leaders with a fast algorithm to calculate the alignment state of the organization.

Quantum computing is a novel computing paradigm that could be useful for this purpose. In quantum computing, information flow and processing are considered to be physical phenomena governed by the laws of quantum mechanics. It is possible because quantum computing makes use of “superposition”, that is, the ability of quantum computers to be simultaneously in multiple different states [17]. Thus, quantum computing has shown promising performance gains in solving certain problems unattainable for classical computing. Shor’s algorithm [18] and Grover’s algorithm [19] are two paradigmatic examples of quantum superior computational performance when compared to classical algorithms.

Guiding an organization toward the coordinated accomplishment of strategic objectives is a probabilistic process in which decision makers can never be sure that the choice made is the right one. Decision-makers are conditioned by the simultaneous decisions of other actors in the organization whose consequences cannot be fully foreseen a priori. Consequently, these networks can be considered decision networks or acyclic probabilistic directed graphical models [20] with known probabilities of alignment. As with the aforementioned genetic algorithm approach, the implementation of this problem as a Bayesian network is computationally very expensive in the presence of a large number of nodes.

**Figure 1:**
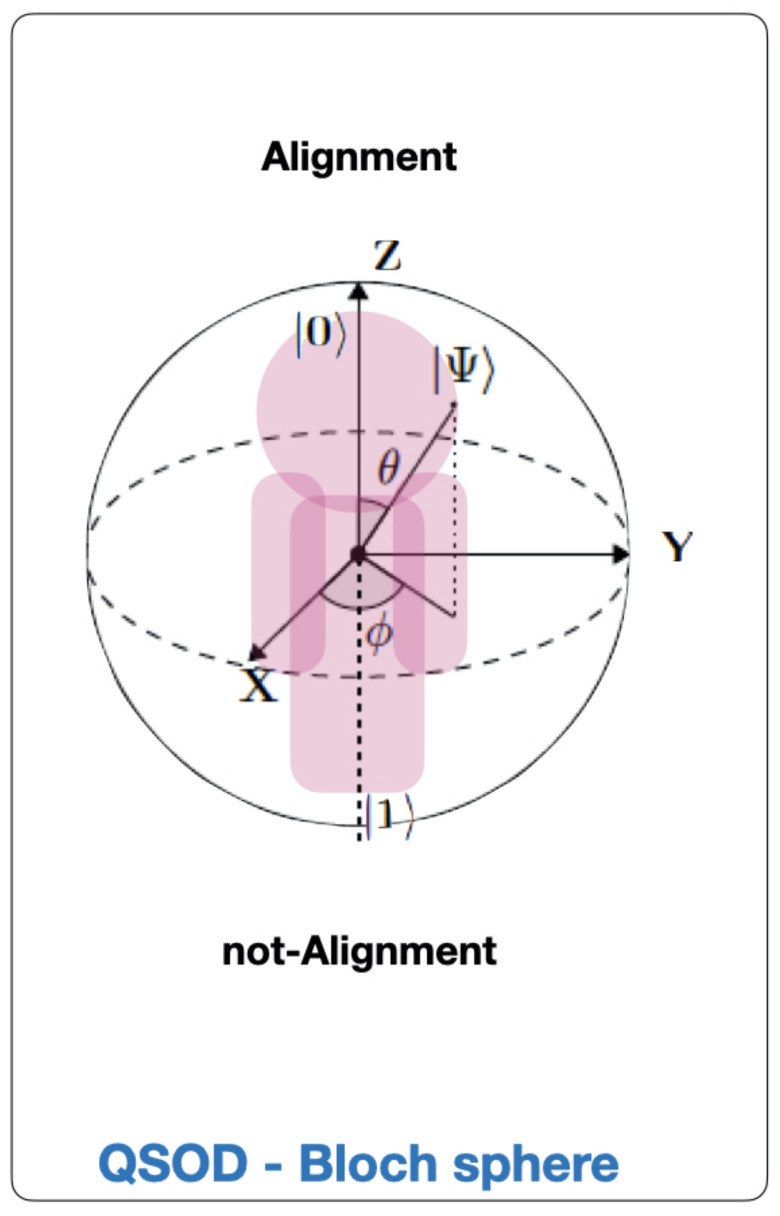
Case study framework.

This work is written for organizational leaders and is designed as a brief disclosure of a significant new application of previous work on quantum strategic organizational design (QSOD) [21,22] which the interested reader should refer to as a framework. Within this framework, QSOD allows for real-time modeling of the states of organizational alignment of complex systems in Industry 4.0. The simulation of QSOD as decision networks and their equivalent quantum circuits opens, without a doubt, a wide field of possibilities for the study of the design of complex strategic networked organizations. As represented schematically in Figure 1, in this work we represent the individual process owner, a complex network node in Industry 4. 0 represented in the form of a decision graph [20], as a quantum computing unit or *qubit* [23,24]. This qubit is allowed to have two fundamental states, one of alignment or asymptotic stability of the key performance indicators (KPIs) defining its performance [2,25,26,27,28,29], represented by the state |0〉 and another of non-alignment, absence of such stability, represented by the state |1〉.

**Figure 2:**
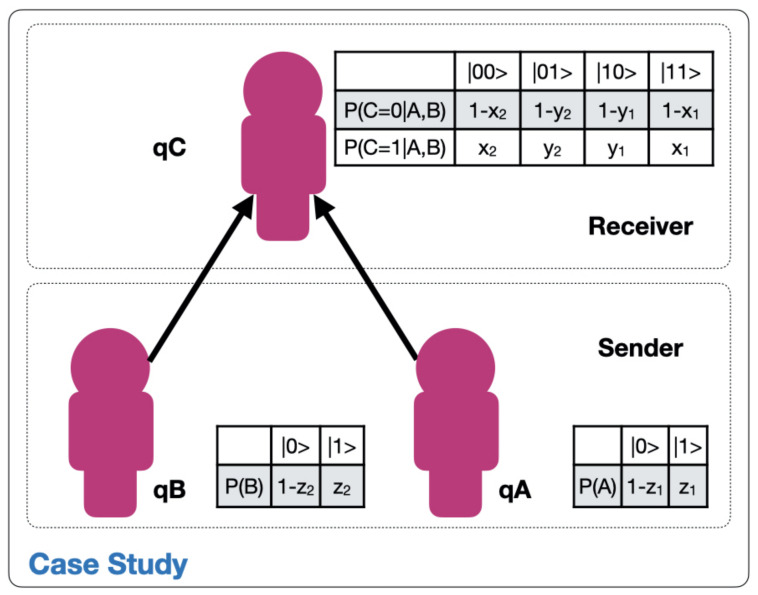
Quantum strategic organizational design (QSOD)-Bloch sphere.

In the previous work [22] we showed how the interaction between two agents, an industrial leader and a subordinate reporting to him, can be interpreted as a dissipative oscillatory system in underdamped mode. In this work, we add a node to this configuration. As shown in Figure 2, we will investigate the case of two subordinate (sender) agents *A* and *B*, reporting simultaneously to a (receiver) leader *C*. As in the case above, the sender and receiver organizational agents are simulated by means of a three-qubit quantum circuit. We aim to investigate the leader’s probability of alignment with the strategic objectives of the organization, depending on the state of his subordinates and their respective probabilities of alignment between them.

The Bloch sphere shown in Figure 1 is the standard qubit geometric representation [30]. The *Z*-axis of Bloch’s sphere, of unitary radius, is the calculation axis and its positive direction coincides with the state |0〉, and the negative with the state |1〉. The state of a qubit given by |Ψ〉 can be represented as a point on the Bloch sphere with the help of two parameters (θ, ϕ), as expressed by Equation (1):

(1) $$|\Psi \rangle =cos\left(\frac{\theta }{2}\right)|0\rangle +e^{i\phi }sin\left(\frac{\theta }{2}\right)|1\rangle$$

Our objective is to establish the alignment probability of agent *C*, P(C=|0〉), as a function of the alignment probabilities of agents *A* and *B* and the alignment probabilities between agent *C* and agents *A* and *B*. This is accomplished by simulating hundreds of different quantum circuit configurations. We present in this work significant findings on the alignment probabilities of the highest-ranking agent depending on the alignment state of their lower rank subordinates.

The rest of the work hereinafter continues as follows: First Section 2 begins with a description of the configuration of the quantum circuit computations necessary to simulate the outlined 3-qubit organizational design configuration. Second, Section 3 presents the case study that will simulate numerous quantum circuits, varying the mentioned parameters in order to obtain an optimal configuration of them. Third, in Section 4 we discuss the results obtained and propose an interpretation from perspective of previous studies and of the working hypotheses. Finally, in Section 5 we discuss the findings and their implications in a broad context, and future research directions and limitations are highlighted.

## 2. QSOD Circuit—3 Qubit Organizational Design Configuration. Two Report to One

In this case, as shown in Figure 2, we will represent a three-qubit system. As explained in [21,23], this requires the use of an additional ancilla-qubit $q^{*}$, whose state is given by $|\Psi^{*}\rangle$, that will allow us to use certain quantum operations that would otherwise be unfeasible. As a consequence, we are faced with a four qubit system whose aggregate state can be expressed as the tensorial product of the individual qubits. The multiple qubit state can be expressed as a linear combination of the |0〉 and |1〉 states, then the aggregated state can be represented as in Equation (2).

(2) $$\begin{array}{cc} |\Psi \rangle & =|\Psi_{A}\rangle ⊗|\Psi_{B}\rangle ⊗|\Psi_{C}\rangle ⊗|\Psi^{*}\rangle =\\ \; & =a_{0}b_{0}c_{0}d_{0}|0000\rangle +a_{0}b_{0}c_{0}d_{1}|0001\rangle +a_{0}b_{0}c_{1}d_{0}|0010\rangle +a_{0}b_{0}c_{1}d_{1}|0011\rangle +\\ \; & +a_{0}b_{1}c_{0}d_{0}|0100\rangle +a_{0}b_{1}c_{0}d_{1}|0101\rangle +a_{0}b_{1}c_{1}d_{0}|0110\rangle +a_{0}b_{1}c_{1}d_{1}|0111\rangle +\\ \; & +a_{1}b_{0}c_{0}d_{0}|1000\rangle +a_{1}b_{0}c_{0}d_{1}|1001\rangle +a_{1}b_{0}c_{1}d_{0}|1010\rangle +a_{1}b_{0}c_{1}d_{1}|1011\rangle +\\ \; & +a_{1}b_{1}c_{0}d_{0}|1100\rangle +a_{1}b_{1}c_{0}d_{1}|1101\rangle +a_{1}b_{1}c_{1}d_{0}|1110\rangle +a_{1}b_{1}c_{1}d_{1}|1111\rangle \end{array}$$

where:
$|\Psi_{A}\rangle =a_{0}|0\rangle +a_{1}|1\rangle$$a_{i}\in C^{2}$$|\Psi_{B}\rangle =b_{0}|0\rangle +b_{1}|1\rangle$$b_{i}\in C^{2}$$|\Psi_{C}\rangle =c_{0}|0\rangle +c_{1}|1\rangle$$c_{i}\in C^{2}$$|\Psi^{*}\rangle =d_{0}|0\rangle +d_{1}|1\rangle$$d_{i}\in C^{2}$

Thus it can be said that the quantum system of 4 qubits can be described by a $2^{4}$-dimensional complex unit vector $|\Psi \rangle \in {\left(C^{2}\right)}^{⊗4}$.

An initial hypothesis of this work is that the leader of the Industry 4.0 organization benefits from knowing its alignment status with the strategic objectives of the organization. That is why we will focus on finding answers to the question of how to maximize the probability of alignment of node *C*, P(C=|0〉), depending on the individual alignment probabilities of the root nodes *A* and *B*, as well as their respective relative probabilities between the nodes given by:

- $1-z_{1}=P\left(A=|0\rangle \right)=1-P\left(A=|1\rangle \right)$. Probability of alignment of node *A*.
- $1-z_{2}=P\left(B=|0\rangle \right)=1-P\left(B=|1\rangle \right)$. Probability of alignment of node *B*.
- $x_{1}=P\left(C=|1\rangle |A,B=|11\rangle \right)$. Probability of no-alignment of node *C* conditioned to the state |11〉 of the waveform $|\Psi_{A}\rangle ⊗|\Psi_{B}\rangle$.
- $y_{1}=P\left(C=|1\rangle |A,B=|10\rangle \right)$. Probability of no-alignment of node *C* conditioned to the state |10〉 of the waveform $|\Psi_{A}\rangle ⊗|\Psi_{B}\rangle$.
- $x_{2}=P\left(C=|1\rangle |A,B=|00\rangle \right)$. Probability of no-alignment of node *C* conditioned to the state |00〉 of the waveform $|\Psi_{A}\rangle ⊗|\Psi_{B}\rangle$.
- $y_{2}=P\left(C=|1\rangle |A,B=|01\rangle \right)$. Probability of no-alignment of node *C* conditioned to the state |01〉 of the waveform $|\Psi_{A}\rangle ⊗|\Psi_{B}\rangle$.

Mathematically speaking, we intend to find the values of $\left(x_{1},y_{1},x_{2},y_{2},z_{1},z_{2}\right)$ that deliver the maximum alignment of node *C* given by Equation (3):

(3) $$\begin{array}{cc} P(C=|0\rangle ) & =f(x_{1},y_{1},x_{2},y_{2},z_{1},z_{2})=\\ \; & =\left|\right|a_{0}b_{0}c_{0}d_{0}{\left|\right|}^{2}+\left|\right|a_{0}b_{0}c_{0}d_{1}{\left|\right|}^{2}+\\ \; & +\left|\right|a_{0}b_{1}c_{0}d_{0}{\left|\right|}^{2}+\left|\right|a_{0}b_{1}c_{0}d_{1}{\left|\right|}^{2}+\\ \; & +\left|\right|a_{1}b_{0}c_{0}d_{0}{\left|\right|}^{2}+\left|\right|a_{1}b_{0}c_{0}d_{1}{\left|\right|}^{2}+\\ \; & +\left|\right|a_{1}b_{1}c_{0}d_{0}{\left|\right|}^{2}+\left|\right|a_{1}b_{1}c_{0}d_{1}{\left|\right|}^{2} \end{array}$$

We will base on the principles of quantum circuit design exposed in [21], to present the quantum circuit that represents the interactions of the decision network exposed in Figure 2 expressed by Equation (4):(4)
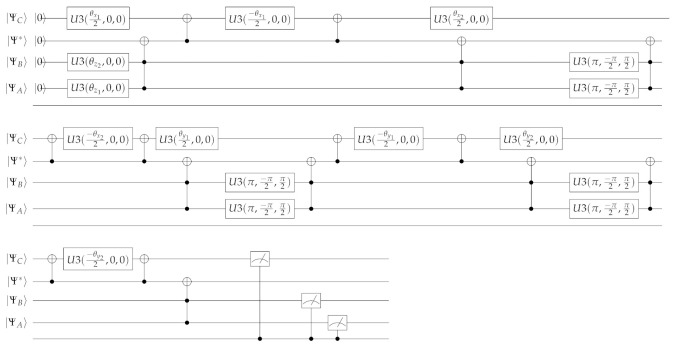


In which the $U_{3}\left(\theta ,\phi ,\lambda \right)$ gate is a single qubit gate that has three parameters θ, ϕ and λ which represent a sequence of rotations around the Bloch sphere’s axes such that [ϕ−π/2] around the *Z* axis, [π/2] around the *X* axis, [π−θ] around the *Z* axis, [π/2] around the *X* axis, and a [λ−π/2] around the *Z* axis. It can be used to obtain any single qubit gate. Equation (5) provides its mathematical representation,

(5) $$U_{3}|\Psi \rangle =\left[\begin{array}{cc} cos(\frac{\theta }{2}) & -e^{i\lambda }sin\left(\frac{\theta }{2}\right)\\ e^{i\phi }sin\left(\frac{\theta }{2}\right) & e^{i(\phi +\lambda )}cos\left(\frac{\theta }{2}\right) \end{array}\right]|\Psi \rangle$$

and Equation (6) its quantum circuit equivalent: (6)
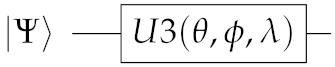


This circuit in Equation (4) presents four qubits $|\Psi_{A}\rangle$, $|\Psi_{B}\rangle$, $|\Psi_{C}\rangle$, $|\Psi^{*}\rangle$ which are rotated through quantum operators. The respective interpretation of these rotations and the equations to calculate them are described in Table 1.

## 3. Case Study

In order to ensure replicability and validation of the results obtained, the source code for the simulations is available under the Open Access Repository (https://osf.io/vzhpg/?view_only=0f890f0f93e3487390cb3d8a6774fc40, accessed on 1 April 2021) which was created with Jupyter Lab Version 1.2.6.

In this case, we are going to proceed to the simulation of quantum circuits that allow elucidating which is the combination of rotations (probabilities) that provides a maximum alignment of the node *C*, given by P(C=|0〉). As shown in Equation (3), the function $P\left(C=|0\rangle \right)=f\left(x_{1},y_{1},x_{2},y_{2},z_{1},z_{2}\right)$ depends on six parameters and a brute-force search with 10% incremental intervals, as for example was done in [22], would be very costly computationally. That is why we are forced to supervise the search algorithm, limiting the parameters to certain plausible intervals where we know the maximum can be found. The first observation in this sense is that the network presents symmetry. Figure 2 shows that as far as node *C* is concerned, nodes *A* and *B* are positioned symmetrically and at the same distance. This allows us to say that the search field can be reduced considerably. Furthermore, we know from [22] that the probability of alignment of a superior node is bounded by the probability of alignment of its subordinate. As a consequence, due to the network’s symmetry, it can be hypothesized that the probability of alignment of node *C* after alignment, $P(C_{post}=|0\rangle )$, has an upper bound given by $\bar{z}$ the mean alignment probabilities of its subordinates, P(A=|0〉) and P(B=|0〉), given by Equation (7):

(7) $$\begin{array}{c} \bar{z}=\frac{\left(1-z_{1}\right)+\left(1-z_{2}\right)}{2}\overset{!}{<}P\left(C_{post}=|0\rangle \right) \end{array}$$

Finally, taking this into account, and given that the probability $P(C_{post}=|0\rangle )$ lower than a random process is not of interest, and we are going to study only values of $\bar{z}$ that are bigger than 0.5.

**Figure 3:**
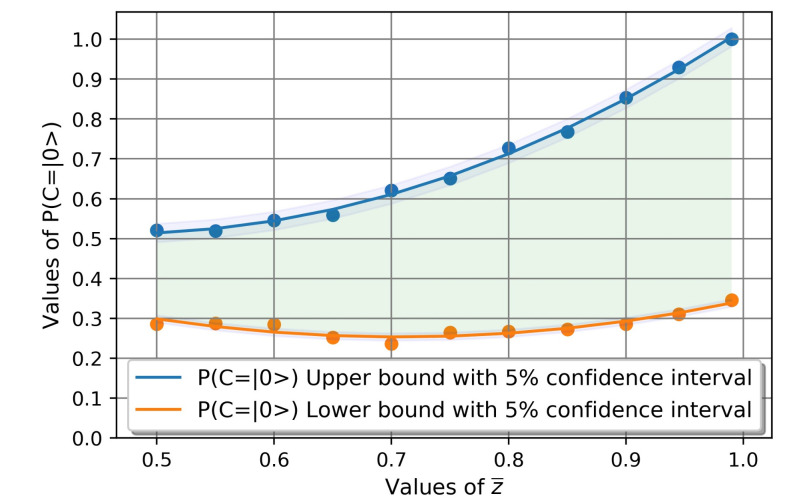
$P(C_{post}=|0\rangle )$ lower and upper bound for different values of $\bar{z}\in \left[0.5,1\right]$.

Taking into account these premises, we have made more than 400 quantum circuit simulations for fixed values of $\bar{z}\in \left[0.5,1\right]$ and numerous values of $\left[x_{1},y_{1},x_{2},y_{2}\right]\in \left[0,1\right]$. The results, together with a polynomial regression curves, are shown in Figure 3. These regression curves are represented with a 5% confidence interval that resemble the uncertainties associated with quantum circuit calculations. The regression curve that fits the upper bound for $P(C_{post}=|0\rangle )$ and its R-squared $\bar{R^{2}}$ factor is described by Equation (8):

(8) $$\begin{array}{cc} \bar{P}\left(C_{post}=|0\rangle \right) & =1.7915{\bar{z}}^{2}-1.667\bar{z}+0.9\;;\;\bar{z}\in \left[0.5,1\right]\\ \bar{R^{2}} & =0.997 \end{array}$$

The regression curve that fits the lower bound for $P(C_{post}=|0\rangle )$ and its R-squared $\underline{R^{2}}$ factor is described by Equation (9):

(9) $$\begin{array}{ccc} \underline{P}\left(C_{post}=|0\rangle \right) & =\bar{P}\left(C_{post}=|0\rangle \right) & =1.061{\bar{z}}^{2}-1.489\bar{z}+0.78\;;\;\bar{z}\in \left[0.5,1\right]\\ \underline{R^{2}} & =0.866 \end{array}$$

**Figure 4:**
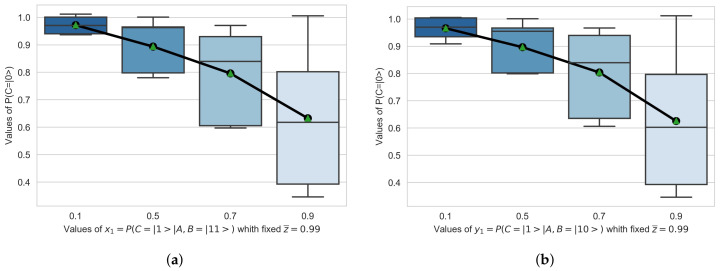
Results obtained for $P(C_{post}=|0\rangle )$, with a fixed $\bar{z}=0.99$, for values of $[x_{1},y_{1})x_{2},y_{2}]\in \left[0.1,0.3,0.5,0.9\right]$. (**a**) $x_{1}=P\left(C_{post}=|1\rangle |A,B=|11\rangle \right)$ (**b**) $y_{1}=P\left(C_{post}=|1\rangle |A,B=|10\rangle \right)$

**Figure 5:**
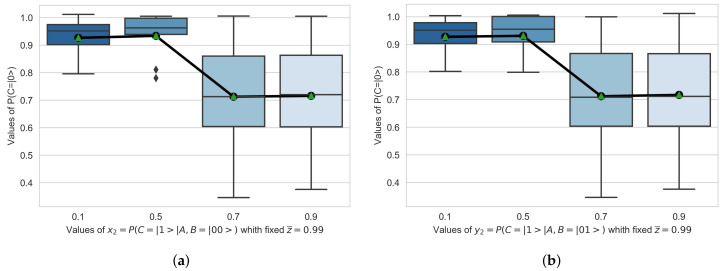
Results obtained for $P(C_{post}=|0\rangle )$, with a fixed $\bar{z}=0.99$, for values of $[x_{1},y_{1})x_{2},y_{2}]\in \left[0.1,0.3,0.5,0.9\right]$. (**a**) $x_{2}=P\left(C_{post}=|1\rangle |A,B=|00\rangle \right)$ (**b**) $y_{2}=P\left(C_{post}=|1\rangle |A,B=|01\rangle \right)$

The green area in Figure 3 includes the entire search spectrum for different values of $\left[x_{1},y_{1}\right),x_{2},y_{2}]\in \left[0,1\right]$. In Figure 4 we represent the values of $P(C_{post}=|0\rangle )$, with a fixed $\bar{z}=0.99$, for values of $\left[x_{1},y_{1}\right)]\in \left[0.1,0.3,0.5,0.9\right]$. In Figure 5 we represent the values of $P(C_{post}=|0\rangle )$, with a fixed $\bar{z}=0.99$, for values of $\left[x_{2},y_{2}\right)]\in \left[0.1,0.3,0.5,0.9\right]$.

In Section 4 we discuss these results in detail.

## 4. Discussion

We will now proceed to discuss the results R systematically. We will start by discussing Figure 3 that describes the relationship between the average alignment probability $\bar{z}\in \left[0.5,1\right]$ of the subordinate nodes *A* and *B* with the alignment probability of the upper node *C*, $P(C_{post}=|0\rangle )$. In this way, the following results can be summarized:

R1. As hypothesized in Equation (7), the alignment probability of node *C* is never greater than the mean alignment probability of its subordinate nodes *A* and *B* given by $\bar{z}\in \left[0.5,1\right]$. This means that in the presented configuration of two nodes reporting to a third one, the node being reported to can never reach a higher alignment probability than those presented by its subordinates.

R2. The amplitude of possible alignment states of node *C*, increases with increasing values of $\bar{z}\in \left[0.5,1\right]$ and is obtained by subtracting Equations (8) and (9). The green shaded area indicates the possible values of this probability, which will be obtained by varying the coefficients $\left[x_{1},y_{1},x_{2},y_{2}\right]$ as already indicated.

R3. In Figure 4 and Figure 5 we indicate, by means of a boxplot, how the alignment probability $P(C_{post}=|0\rangle$ behaves within its bounds. In both cases we observe how this probability has a lower bound. In Figure 4 this lower bound is given by the relative probability of alignment of $P(C_{post}=|0\rangle$, conditioned to the states $x_{1}=P\left(C_{post}=|1\rangle |A,B=|11\rangle \right)$ and $y_{1}=P\left(C_{post}=|1\rangle |A,B=|10\rangle \right)$ respectively. This means that given $x_{1}=P\left(C_{post}=|1\rangle |A,B=|11\rangle \right)$ or $y_{1}=P\left(C_{post}=|1\rangle |A,B=|10\rangle \right)$ are in the indicated states, the probability of alignment $P(C_{post}=|0\rangle$ is equal or bigger. In Figure 5 this lower bound is given by the relative probability of alignment of $P(C_{post}=|0\rangle$, conditioned to the states $x_{2}=P\left(C_{post}=|1\rangle |A,B=|00\rangle \right)$ and $y_{2}=P\left(C_{post}=|1\rangle |A,B=|01\rangle \right)$ respectively. This means that given $x_{2}=P\left(C_{post}=|1\rangle |A,B=|00\rangle \right)$ or $y_{2}=P\left(C_{post}=|1\rangle |A,B=|01\rangle \right)$ are in the indicated states, the probability of alignment $P(C_{post}=|0\rangle$ is equal or bigger.

In the following Section 5 we offer the conclusions derived from these results, we discuss the findings and their implications in a broad context, offer certain limitations of the study, and present possible next research paths to pursue.

## 5. Conclusions, Limitations and Further Steps

We can formulate the most important conclusion of this work, derived from R1, with the following statement: The alignment probability of a node to which two nodes report cannot be greater than the average of the alignment probability of these. In other words, the alignment probability of a boss can never be greater than the average of the alignment probability of his two subordinates. The implications of this are very powerful and relevant for leaders and organizational design scholars alike. On the one hand, this means that in order to increase the level of organizational hierarchies and preserve asymptotic stability towards the organizational strategic objectives, and therefore the low levels of associated variability, it is necessary that the lower levels present such a high or superior stability. This seems to indicate that we can only expand an organization to higher levels of complexity by adding new hierarchical layers if we have achieved high levels of stability at the lower levels. This fact is in accordance with previous results presented in [22,31].

In R2 we observe that increasing the average probability of alignment of the lower nodes, increases the probability of alignment of the upper node. This is in accordance with the results obtained previously in [22] of one node reporting to another. Moreover, $x_{1}=P\left(C_{post}=|1\rangle |A,B=|11\rangle \right)$=$y_{1}=P\left(C_{post}=|1\rangle |A,B=|10\rangle \right)$ and $x_{2}=P\left(C_{post}=|1\rangle |A,B=|00\rangle \right)$=$y_{2}=P\left(C_{post}=|1\rangle |A,B=|01\rangle \right)$ then we have the case of a perfectly aligned node, and the problem is reduced to the case presented in [22] of one node reporting to another.

Likewise, if we compare the results shown in Figure 4 and Figure 5 presented in R3 with the results obtained in [22] that show the case of a node reporting to another one, it can be inferred that the addition of a new node reporting to the superior node adds stability to the set. In other words, the harmonic underdamped oscillation that was observed between the alignment states in the case of one node reporting to another, has disappeared in the case of two nodes reporting to a third. This seems to indicate that the additional node provides additional stability to the organizational system.

The main limitation of this study is that it only refers to one configuration of all possible configurations involving three agents. Furthermore, the simulations of the quantum circuits have been made in a classic computer simulator. While this undoubtedly reduces some statistical significance to the results, this fact is not relevant to our study at this time and can be neglected.

The results obtained studying the QSOD case of 3 qubits, in which two reports to another, opens new interesting research questions. In order to continue offering a valuable contribution to Industry 4.0 leaders and the research community in general, the future steps we intend to take in this line of research will focus on studying the behavior of other 3 qubits QSOD configurations.

This work has been presented as part of a whole and describes a concrete but very relevant motif often found in organizations: The simultaneous reporting of two elements to another. The scientific relevance of this article lies in its multiple applications. While it is true that the application case has been proposed for a very specific case of application that describes the relationship between three agents in an Industry 4.0 environment, the same QSOD logic can be applied to other relevant cases. For example, to cite a few, the competitive relationship that exists between two suppliers to a customer, where the relative probabilities of the qubits could refer to the delivery capacity, or applied to the field of project management, could be understood as the probability of completion of two resources on which a third party depends. In general, the logic presented by the QSOD can be applied to any decision network. This work and those related presented above [21,22] invite such a generalization that presumably will open new frontiers in knowledge, since it combines quantum simulations with management for the first time.

## Figures and Tables

**Table 1:** Qubit angles of rotation.

Qubit	Interpretation	Equation
$|\Psi_{A}\rangle$	The probability $z_{1}=P\left(A=|1\rangle \right)$ of qubit $|\Psi_{A}\rangle$ to be in non-alignment translates into the rotation angle $\theta_{z_{1}}$.	$\theta_{z_{1}}=2\arctan\sqrt{\frac{z_{1}}{1-z_{1}}}$
$|\Psi_{B}\rangle$	The probability $z_{2}=P\left(B=|1\rangle \right)$ of qubit $|\Psi_{B}\rangle$ to be in non-alignment translates into the rotation angle $\theta_{z_{2}}$.	$\theta_{z_{2}}=2\arctan\sqrt{\frac{z_{2}}{1-z_{2}}}$
$|\Psi_{C}\rangle$	The probability $x_{1}=P\left(C=|1\rangle |A,B=|11\rangle \right)$ of qubit $|\Psi_{C}\rangle$ to be in non-alignment depending on the probability of the waveform $|\Psi_{A}\rangle ⊗|\Psi_{B}\rangle$ to be in the state |11〉 translates into rotation angle $\theta_{x_{1}}$.	$\theta_{x_{1}}=2\arctan\sqrt{\frac{x_{1}}{1-x_{1}}}$
	The probability $y_{1}=P\left(C=|1\rangle |A,B=|10\rangle \right)$ of qubit $|\Psi_{C}\rangle$ to be in non-alignment depending on the probability of the waveform $|\Psi_{A}\rangle ⊗|\Psi_{B}\rangle$ to be in the state |10〉 translates into rotation angle $\theta_{y_{1}}$.	$\theta_{y_{1}}=2\arctan\sqrt{\frac{y_{1}}{1-y_{1}}}$
	The probability $x_{2}=P\left(C=|1\rangle |A,B=|00\rangle \right)$ of qubit $|\Psi_{C}\rangle$ to be in non-alignment depending on the probability of the waveform $|\Psi_{A}\rangle ⊗|\Psi_{B}\rangle$ to be in the state |00〉 translates into rotation angle $\theta_{x_{2}}$.	$\theta_{x_{2}}=2\arctan\sqrt{\frac{x_{2}}{1-x_{2}}}$
	The probability $y_{2}=P\left(C=|1\rangle |A,B=|01\rangle \right)$ of qubit $|\Psi_{C}\rangle$ to be in non-alignment depending on the probability of the waveform $|\Psi_{A}\rangle ⊗|\Psi_{B}\rangle$ to be in the state |01〉 translates into rotation angle $\theta_{y_{2}}$.	$\theta_{y_{2}}=2\arctan\sqrt{\frac{y_{2}}{1-y_{2}}}$
$|\Psi^{*}\rangle$	The ancilla qubit $|\Psi^{*}\rangle$ is a support qubit and as such is not subject to any probability rotation.	

## Abbreviations

The following abbreviations are used in this manuscript:

QSOD	Quantum Strategic Organizational Design
KPI	Key Performance Indicators
